# Guiding principles for quality, ethical standards and ongoing learning in implementation research: multicountry learnings from participatory action research to strengthen health systems

**DOI:** 10.1093/heapol/czaa123

**Published:** 2020-11-06

**Authors:** Kim Ozano, Laura Dean, Oluwatosin Adekeye, Anthony K Bettee, Ruth Dixon, Ntuen Uduak Gideon, Noela Gwani, Sunday Isiyaku, Karsor Kollie, Luret Lar, Akinola Oluwole, Helen Piotrowski, Alice Siakeh, Rachael Thomson, James Yashiyi, Georgina Zawolo, Sally Theobald

**Affiliations:** 1 Liverpool School of Tropical Medicine, Pembroke Place, Liverpool L35QA, UK; 2 Sightsavers, Nigeria Country Office, Golf Course Road, City Centre, Kaduna, Nigeria; 3 Ministry of Health, Government of Liberia, SKD Boulevard, Monrovia, Liberia; 4 Sightsavers, 35 Perrymount Rd, Haywards Heath RH16 3BZ, UK; 5 Ministry of Health, Government of Nigeria, Federal Secretariat, Complex Garki PMB, 83 Abuja, Nigeria; 6 Pacific Institute for Research and Evaluation UL-PIRE Africa Center, University of Liberia, Capitol Hill Liberia

**Keywords:** Implementation research, participatory action research, quality, ethical standards, health systems strengthening

## Abstract

Global health gains can be achieved through strengthening health systems to identify and address implementation challenges in low- and middle-income countries. Participatory research, that promotes joint problem and solution finding between communities and different health systems actors, supports policy implementation analysis at all levels. Within the neglected tropical disease programmes in Liberia and Nigeria, we applied participatory action research (PAR) to address programmatic and health system bottlenecks with health systems strengthening embedded. This paper shares learning from 20 interviews with co-researchers, from national and sub-national levels and academic researchers who worked collaboratively to understand challenges, co-create solutions and advocate for policy change. Through analysis and reflections of existing PAR principles, we inductively identified five additional guiding principles for quality, ethical standards and ongoing learning within PAR projects that aim to strengthen health systems. (1) Recognize communities as units of identity and define stakeholder participation to ensure equitable engagement of all actors; (2) enable flexible action planning that builds on existing structures whilst providing opportunities for embedding change; (3) address health systems and research power differentials that can impede co-production of knowledge and solution development; (4) embed relational practices that lead to new political forms of participation and inquiry within health systems and (5) develop structures for ongoing learning at multiple levels of the health system. PAR can strengthen health systems by connecting and co-creating potentially sustainable solutions to implementation challenges. Additional research to explore how these five additional principles can support the attainment of quality and ethical standards within implementation research using a PAR framework for health systems strengthening is needed.

**KEY MESSAGES**

- Participatory action research as an implementation framework strengthens health systems and supports researchers, implementers, policy makers and communities to connect and co-create sustainable solutions to implementation challenges.
- The values and principles of participatory action research, when applied to address implementation challenges, allow for the negotiation of power between and across health systems actors and communities, promoting a bottom-up approach to tackling implementation challenges.
- Five key principles (identified here) can support the assessment of quality and ethical standards within implementation research projects where health systems strengthening is also a central aim.
- Action learning approaches that consider scale up across multiple levels of the health system ensures solutions to implementation challenges are co-created, embedded and sustained.

## Introduction

### Implementation research–participatory action research as a framework

Implementation research frameworks are applied across diverse settings, sectors and disciplines within global health to strengthen the delivery of public health policies and services (Theobald *et al.*, 2018). The increasing demand for real-world, context-specific solutions to implementation problems has led to a growing interest in the co-production of health systems knowledge and learning (Di Ruggiero and Edwards, 2018; D’Ambruoso *et al.*, 2019).

One framework, applied within implementation research is participatory action research (PAR) which is gaining traction as a mechanism for strengthening health systems and improving health programme delivery (Tetui *et al.*, 2017a; Di Ruggiero and Edwards, 2018; Martineau *et al.*, 2018; Tetui *et al.*, 2018). PAR is a cyclical research process of problem identification, action and reflection leading to further inquiry and action for change through democratic processes of decision making (Kindon *et al.*, 2007; Chevalier and Buckles, 2013; Di Ruggiero and Edwards, 2018). PAR core principles include; respect for diversity, community strengths, reflection of cultural identities, power-sharing and co-learning (Minkler, 2000). In low- and middle-income countries (LMICs) participatory research is often centred at the community level rather than across the multiple levels in which health systems operate, with fewer examples of PAR at National, State and Regional levels (Haaland and Vlassoff, 2001; Keusch *et al.*, 2010; Tetui *et al.*, 2017a). When the PAR approach has been applied to strengthen health systems and address implementation challenges at the sub-national level, ownership and long-term sustainability of interventions has been demonstrated (Tetui *et al.*, 2017b; Martineau *et al.*, 2018) illustrating its potential for change.

A review examining what is required for health systems strengthening (HSS), highlights key elements to consider; scope, scale, sustainability and effects of health outcomes, equity and responsiveness (Witter *et al.*, 2019). However little research has explored the intersection of the science and practice of participatory approaches that have the potential to simultaneously strengthen health systems whilst addressing implementation challenges (Wallerstein and Duran, 2010; D’Ambruoso *et al.*, 2019). Here, we explore how PAR is an opportunity to help researchers, implementers and communities to connect and address implementation challenges (Mshelia *et al.*, 2013; Di Ruggiero and Edwards, 2018; D’Ambruoso *et al.*, 2019) using neglected tropical disease (NTD) programmes as an example.

### Quality and ethical standards in implementation research frameworks

PAR approaches have been used in many global health programmes but recent debates about what constitutes ‘scientific rigour’ and ethical standards in implementation research has led to questions of validity and quality within what is often described as a ‘messy’ research process (Lehmann and Gilson, 2015). Scientific design in quantitative or more structured qualitative research methodologies have clear ethical standards and quality measures that have been established through time. Within PAR the cyclical, collaborative nature of decision-making means that health systems actors (HSAs) and communities drive the process, thus the intervention content is mostly unpredictable and evolves after ethics has been gained. This does not mean that ethical standards, trustworthiness and quality are not essential but rather brings complexity and requires different mechanisms to demonstrate scientific rigour (Springett *et al.*, 2011). Yet, literature exploring the processes that make PAR ethical and of quality whilst remaining scientific are less common than those reporting outcomes of PAR research.

Multiple groups and authors have developed various principles and models to ensure and measure ethical standards and quality in PAR (Banks *et al.*, 2013; Belone *et al.*, 2016; Springett *et al.*, 2016; Israel *et al.*, 2017). One of the mechanisms applied by PAR researchers to measure quality and ensure ethical standards is to certify that related principles are adhered to and documented throughout the research process. For example, Israel *et al.* (2017) identify 10 core principles (Box 1) of participatory research. The International Collaboration of Participatory Health Research (ICPHR) has developed a position paper entitled ‘Ensuring Quality: Indicative Characteristics of Participatory (Health) Research’ which have 11 quality principles. Springett *et al.* (2016) identified 15 categories of description which represent the different ways that quality in participatory research is understood. Belone *et al.* (2016) developed a conceptual/logic model of participatory research partnerships to understand the contribution of partnership processes to improved community capacity and health outcomes. However, these principles do not necessarily have HSS as a core aim. Therefore, we collected reflections from research partners at all levels and analysed these using PAR principles as a quality and ethical standards framework, whilst also allowing for inductive identification of any new principles that may be more aligned to HSS. Our multi-disciplinary background with expertise in both HSS and PAR approaches added trustworthiness to the process and allowed for cross validation of new principles. These principles will add quality and promote critical analysis of relational dynamics within PAR projects for HSS within implementation research.

**Box 1 Principles of community-based participatory research from Israel *et al.* (2017)**

1. Recognizes communities as a unit of identity
2. Integrates and achieves a balance between research and action for the mutual benefit of all partners
3. Involves systems development through a cyclical and iterative process
4. Builds on strengths and resources in the community
5. Facilitates collaborative, equitable partnerships in all research phases and involves an empowering and power-sharing process that attends to social inequalities
6. Promotes co-learning and capacity building among all partners
7. Disseminates findings and knowledge gained to all partners and involves all partners in the dissemination process
8. Emphasizes public health problems of local relevance and ecological perspectives that attend to the multiple determinants of health and disease
9. Requires a long-term process and commitment to sustainability
10. Addresses issues of race, ethnicity, racism and social class and embraces ‘cultural humility’

This paper aims to: (1) explore how the principles of PAR can be used to assess quality of implementation research projects designed to strengthen health systems and improve health programmes; (2) discuss how PAR facilitated a means of exploring health system relational dynamics and the processes that support systems and policy change; (3) share and present evidence for five additional principles to support high quality, ethical PAR to address implementation challenges for HSS, developed from implementation research in Liberia and Nigeria.

### The PAR setting

Table 1 explains the overall project scope together with a brief description of the context of Liberia and Nigeria and where each research team is within the PAR cycle. The PAR cycle applied in this research is also shown in Figure 3. Additional information about new community engagement techniques co-designed through the PAR process to address problems identified can be found in the blog by Lar *et al*. (2018).

**Table 1:** Research context

COUNTDOWN: The research is part of the COUNTDOWN research consortium (https://countdown.lstmed.ac.uk/), a 7-year implementation research project which brings together multi-disciplinary NTD researchers, policy makers, practitioners and implementation research specialists. Through implementation research in Liberia and Nigeria the project aims to improve the efficiency, equity and sustainability of current NTD control approaches, to strengthen programmes at the national, district, community and household level. The funding supported only research activities and not implementation of NTD-related activities. Implementation included adaptations proposed to routine NTD implementation identified as part of the PAR process.	
Liberia country context	Nigeria country context
Liberia has a complex socio-political history. A 14-year civil conflict devastated the countries health and social infrastructure and led to widespread extreme poverty. After a short period of reform, in 2014 the Ebola epidemic caused further damage to Liberia’s health system and led to a breakdown in trust between communities and service providers. Liberia is currently in another period of rapid policy reform and reflection. The NTD programme has now resumed interventions after the Ebola epidemic with the aim to catch up with the gains lost as the result of the outbreak ( Ministry of Health, Government of Liberia, 2015; Bell *et al*., 2017; Thomas *et al*., 2017; ,). The NTD programme in Liberia was established in 2011, however, ongoing periods of fragility and disruption have meant that the delivery of NTD services, including community directed treatment is frequently interrupted. To ensure no one is left behind in ongoing service delivery we need to understand the applicability of diverse and innovative approaches that enable minimal disruption to programme delivery during periods of social and political change.	Nigeria is culturally diverse and multi-ethnic, accounting for approximately half of the West Africa Region’s population with one of the largest global youth populations. Despite having an abundance of natural resources, the majority of the population still live in poverty, without access to basic services and a lack of inclusive development policies (The World Bank, 2019). The health system has three tiers of government administration; Federal, State and Local which share responsibilities for providing health services and programmes. Community directed treatment for NTDs has been ongoing for 20 years, however, some populations remain untreated, and questions remain about the ongoing applicability and sustainability of these interventions during periods of programmatic, social, political and environmental change.
Liberia position within PAR cycle	Nigeria position within PAR cycle
The Liberia team are in the action planning phase of the research process. They have: applied participatory methodologies with communities, frontline health workers, county and national health systems staff to co-develop solutions to identified implementation challenges engaged in policy dialogue with key stakeholders at different levels of the health system developed an NTD-specific national communication strategy including an operational plan that links to and informs the national health promotion strategy to enhance cross-departmental collaboration The next step will be to implement and observe co-created action plans.	The Nigeria team have implemented a full PAR cycle. They have: applied participatory methodologies with communities, frontline health workers, LGA, State and national health systems staff to co-develop solutions to identified implementation challenges engaged in policy dialogue with key stakeholders at different levels of the health system produced and co-implemented action plans with implementers at the state and LGA level observed and reflected on the process Revised and adapted action plans The next step is to go through another PAR cycle whilst scaling-up the approach to additional local government areas.

At each phase of the PAR cycle in Liberia and Nigeria we documented critical reflections of researchers and HSAs as they negotiated complex power dynamics. Through interviews (20), we asked them to think critically about the interactions and negotiations they went through to ensure quality ethical practice as they used PAR to co-identify and address implementation challenges in NTD control and elimination. In the following sections we draw on these critical reflections, from across two diverse socio-political contexts, to support the development of key additional guiding principles for quality, ethical standards and ongoing learning in implementation research.

## Methods

### Data collection

Our PAR approach in Liberia and Nigeria involved building relationships and challenging existing power differentials across the health system. Key cross-country reflections about the PAR process were captured in 20 (5 from Liberia and 15 from Nigeria) interviews with researchers and co-researchers (who are also NTD programme implementers). There were fewer interviews in Liberia as they have a smaller research implementation team, as well as fewer health system levels and implementers compared with Nigeria through which to draw health systems stakeholders from. In addition, they are at an earlier stage within the PAR cycle which meant that relationships for implementing action plans were still in development during the writing of this paper and reflexivity from lower level stakeholders (e.g. county and district health team members) in their infancy. Reflexive diaries from lead researchers complement the analysis of interviews and add detail to the challenges faced when implementing PAR. Reflexive diaries were captured either through audio recording of individual or group reflexive sessions which were then transcribed, and some researchers kept written personal reflexive diaries. UK researchers were also part of group reflexive diary processes and had their own reflexive discussions which also fed into the development of the principles. In Liberia group reflexivity sessions were held at the end of each activity, with the lead researchers keeping written notes of observations and reflections of the process.

To ensure the data is as anonymous as possible we have summarized the interviews into categories and will use the nomenclature found in Box 2 within the findings and discussion which are presented together.

**Box 2**

Summary of interview groups and nomenclature within the paper

- ‘Research leads’ are based within UK, Nigeria or Liberia and have social science expertise (6)
- ‘HS Co-Rs' are personnel from Federal/National and State levels who have a key role in NTD programmes and have received PAR training from researchers (5)
- ‘HSAs' are stakeholders engaged in the PAR process but are not a co-researcher in the sense of collecting data using participatory research methods. This includes national and sub-national NTD implementers, medical officers, frontline health facility staff and an NTD research advisor (9)

### Analysis

Interviews and reflexive diaries were recorded and transcribed verbatim. Personal written diaries were submitted by researchers after obtaining consent for their use. Data were analysed thematically using an inductively developed coding framework; applied using NVivo 11. Similarities and differences within each code were reviewed to develop thematic charts with themes and subthemes (Ritchie *et al.*, 2013). Explanatory accounts of the data were produced by comparing and aligning inductively identified themes with PAR principles and other frameworks related to PAR quality and ethical standards (Banks *et al.*, 2013; Belone *et al.*, 2016; Springett *et al.*, 2016; Israel *et al.*, 2017). Finally, we identified five additional principles that could be utilized by projects applying PAR for HSS in LMICs to ensure ethical standards, quality and ongoing learning for addressing implementation challenges. These five principles are presented below alongside an explanatory account of the processes and reflections from interviewees that relate to the principles, and links to the literature on PAR, quality, ethics, learning health systems, HSS and implementation research.

### Ethical considerations

All research processes were approved by the authors’ institutes. Informed consent was obtained from all participants. Some of the research participants are also authors of the paper as they are academic researchers within the PAR programmes.

## Results and discussion

**Figure 1:**
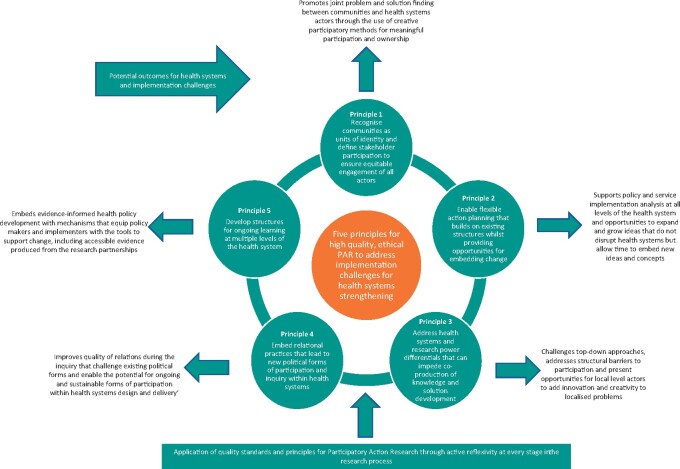
Five principles for high quality, ethical PAR to address implementation challenges for HSS

This section presents the five additional guiding principles inductively identified during research analysis. Within each principle, the links with existing PAR principles are presented followed by a description of how and why the new principle was derived. Then using examples from the data collected in Liberia and Nigeria, and the literature, we reflect on how each principle addressed implementation challenges and added quality to the PAR process. A summary of the five principles and the potential quality outcomes for health systems and implementation challenges is presented in Figure 1.

### Principle 1: Recognize communities as units of identity and define stakeholder participation to ensure equitable engagement of all actors

#### Links to existing PAR principles

**Figure 2:**
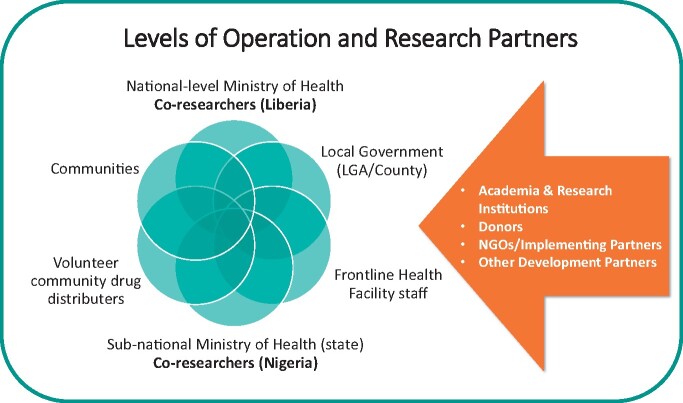
Levels of operation and research partners in health systems

When applying PAR for HSS, there are two overlapping levels of ‘community’; the health system and its actors, and community members that are affected by a health issue (see Figure 2). Community members include people living or working in NTD endemic communities who are eligible to receive NTD preventive chemotherapy annually. The ‘community’ of HSAs are those that have a role to play in planning and implementing mass administration of medicines to the population at different levels of the health system as defined in Box 2. To understand more about the roles of each community and the problems and solutions posed, please refer to the Nigeria learning packs (COUNTDOWN Nigeria, 2018) found online and the two supplementary files entitled; ‘*Participatory guide for planning mass administration of medicines to tackle Neglected Tropical Diseases’* and the ‘*Liberia Neglected Tropical Diseases Communication Strategy 2019-23*.’

Health policy and systems research aims to create useful knowledge that has meaning to the people who make up the system and who can bring about systematic changes in different contexts (Sheikh *et al.*, 2014; D’Ambruoso *et al.*, 2019). PAR principles of inclusivity, shared ownership and valuing the opinions of all presents an ideal space for connecting not only those at the national level of the health system, but those at intermediary levels, health workers and communities. Negotiating existing community hierarchies (gender, age and other) and power differentials within participatory research processes is also essential to ensure that systems of dominance are not reinforced and to promote equitable responses. The development of people centred health systems require that those with the greatest health needs have the most say in ensuring how these needs are met (Sheikh *et al.*, 2014).

This principle therefore adds the dimension of defining stakeholder participation as interviewees reflected on the need to re-orient thinking towards a more participatory worldview to ensure equitable engagement of all actors. This is also endorsed by a recent literature review highlighting mechanisms for HSS where it was identified that synergistic collaboration of stakeholders to achieve long-term strategic reform goals across all health systems levels is key to improving health and health access (Witter *et al.*, 2019). Understanding how different communities would relate to the research was therefore an important consideration for facilitators of PAR amongst both levels of communities to ensure the development of synergistic relationships (D’Ambruoso *et al.*, 2019; Witter *et al.*, 2019).

#### Examples from the research related to units of identity, power and defining stakeholder participation to ensure equitable engagement of all actors

The following examples from the data provide evidence related to this additional principle. We believe this principle added quality to the project and improved understandings of relational dynamics within and between health systems and communities, specifically in relation to power, participation and meaningful community engagement.

Research leads in PAR, as facilitators, described having to bridge longstanding communication gaps between health systems and communities which added complexity in comparison with research projects that work solely at the community level or with HSAs.

To support an atmosphere for co-creation, researchers must constantly build and maintain trust and promote attitudes that reflect dignity, respect and mutuality (Cleary *et al.*, 2018). Karnieli-Miller *et al.* (2009) state the researcher’s role is to create a welcoming, informal, anti-authoritive and non-hierarchical atmosphere in which participants can explore challenges and solutions. The Liberia team reflected on how they nurtured respect and developed relationships with the community;

*I would tell them …we are similar, similar kinds of people and we should respect other people’s views…You have to develop the relationship with your community if you want to have successful research* (Research lead, Liberia).

#### Health system actors as ‘units of identity’ and pre-existing power differentials that affected participation

HSAs are inherently hierarchical with power differentials central to systems functioning (Witter *et al.*, 2019). Recognizing and addressing pre-existing power structures was essential from the onset of the project as dialogical processes involved in PAR are only successful if issues of power are acknowledged and addressed (Springett *et al.*, 2011). To initiate this process, a week-long training was delivered by the academic research team with discussions of how power is negotiated and reflected including considerations of positionality. Co-researchers from the National (Liberia) and sub-national levels (Nigeria) received training and were involved in data collection and analysis. However, in LMICs the level of HSAs participation must include consideration that the HS may already be under-resourced and so ethically a discussion should be had to weigh-up the benefits from participating in the research versus the distraction from delivering services.

#### How power and participation of community members affected by NTDs was negotiated

Recognizing the views and priorities of diverse community members is critical to the PAR process to ensure that the potential roles, capabilities, needs and preferences of all individual actors within communities are acknowledged and prioritized within the operation of health systems activities (Abimbola *et al.*, 2014). A health systems co-researcher (HS Co-R) identified how the PAR approach considered different community groups who are likely to identify a range of solutions;

*They [researchers] take their time by going deep and involving different groups, people, thereby hearing their [community members] own ideas, what they want and the changes they want to see and by so doing there will be different ideas and different solution …* (HS Co-R, Nigeria).

In Liberia and Nigeria, the use of participatory methods helped to relinquish power to community members which served not only to create a more ethical research situation, but also to generate new forms of knowledge which cannot be established any other way (Packard, 2008). The researchers (which included HSAs) had to think critically about their power and acted as facilitators as described by one HS Co-R;

*… we know that they have their own way of giving just as you have your own way of contributing …. They made this research successful. In their absence no one is ever going to get findings* (HS Co-R, Liberia).

#### Strengthening capacity to facilitate meaningful community participation

Community ownership and participation is a key driver for HSS reported in LMICs (Samuels *et al.*, 2017). Research leads highlighted the importance of community participation to address programmatic challenges;

*… So, I mean getting a voice - you allow them to tell you what they think about their situation, to clearly explain to you about their situation, how they want it to be improved and what’s the way forward* (Research lead, Liberia).

To ensure meaningful community participation, a process of critical thinking and analysis is essential to gaining insightful knowledge and creating an empowering environment for open exploration of problems and solutions (Haaland and Vlassoff, 2001; George *et al.*, 2016). A HSA who was also a co-researcher in Liberia spoke of the importance of the communities feeling empowered and taking ownership in the process;

*They are involved in solving their own problems instead of someone coming from outside to tell them what needs to be done… there is ownership in there… You get much more committed in solving that problem than if someone just imposed it on you* (HS Co-R, Liberia).

To enhance community capacity to critically reflect on health programmes and their impacts, participatory methods were applied including transect walks, social mapping, visual story boards that represented key intervention challenges, drawing and a participatory ranking exercise (Dixon and Lar, 2018; Zawolo *et al.*, 2018; COUNTDOWN Consortium, 2019). The Liberia team reflected on how using visual methods with community members enhanced that critical thinking process;

*… using the visual method is where they are able to fully participate and maybe give you more information than just asking ‘what do you think about this?’ …* (Research lead, Liberia).

Local level ownership was a key outcome of being part of the PAR process;

*… normally when I came, I don’t know all the settlement within [LGA]. But through the research, going with them, I now know more than 90% of the community. Then, also, its, let me believe that the program is mine …* (local level HSA, Nigeria).

### Principle 2: Enable flexible action planning that builds on existing structures whilst providing opportunities for embedding change

#### Links to existing PAR principles

**Figure 3:**
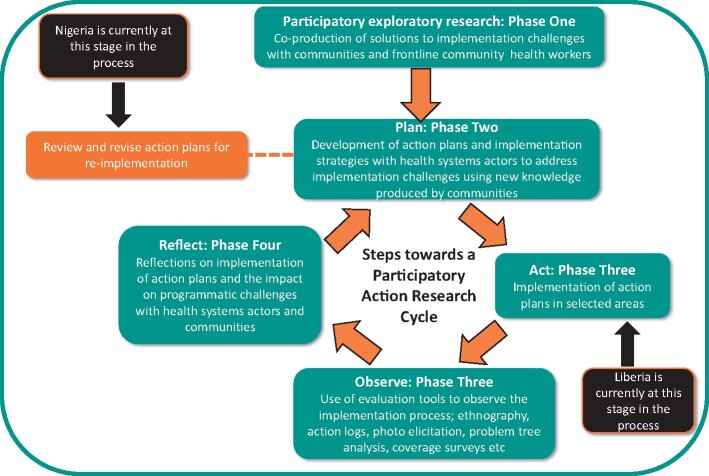
PAR cycle and country position

This principle drew from the CBPR principle by Israel *et al.* (2017) related to systems development through a cyclical and iterative process that involves cycles of co-production, review, revision and implementation with ongoing and dynamic processes of change that respond to context. The cyclical process being applied in each country context is indicated in Figure 3, depicting where each country is in the process. When considering Israel *et al.*’s (2017) principle we found clear evidence of the need for flexibility within the action planning process to support HSS, as well as considering and building on existing structures/constraints within health systems to promote and embed realistic changes.

#### Practical examples of flexible action planning that built on existing structures whilst providing opportunities for embedding change

The following practical examples demonstrate some of factors within health systems that were inductively found to be conducive or restrictive to HSS during action planning including how flexible action planning and consideration of existing structures was useful.

By recognizing and acknowledging existing resources (and restraints), implementation structures and mechanisms which are familiar to communities and HSAs, there are opportunities to expand and grow ideas that do not disrupt health systems but allow time to embed new ideas and concepts. For example, in Nigeria, researchers adapted the planning template usually used by NTD implementers and in Liberia, the communication strategy adopted the existing national template for development of health communication approaches which supported the NTD team to use the national health promotion approach and facilitated cross departmental learning.

One of the risks when developing actions for change is that some solutions may not be supported due to financial, security or other programmatic restrictions;

*… there were instances where we couldn’t carry out activities or research activities as planned … because the environment, people, the implementers, the state, the government unit were not ready for these activities to be carried out at those times …* (Research lead, Nigeria).

We tried to manage this by presenting solutions developed by communities in a flexible ‘shopping list’ or ‘action bundle’ style for selection based on feasibility (COUNTDOWN Consortium, 2019). However, not being able to implement all solutions caused some frustration;

*I would like more of the findings the COU* ***NTD*** *OWN research made in the field to try and encourage the supporting NGOs and the state to correct and implement the challenges encountered in the field* … (HS Co-R, Nigeria).

One of the challenges highlighted within action learning approaches for HS is that the process involves cooperative interactions between different stakeholders that often have distinct expectations, priorities and interests (Lehmann and Gilson, 2015). As action researchers we must be aware of this risk and mitigate against it wherever possible. Initial statements or terms of reference may have served to better manage expectations and will be considered in the future. This is a consideration that would likely support sustainable systems and policy change.

### Principle 3: Address health systems and research power differentials that can impede co-production of knowledge and solution development

#### Links to existing PAR principles

One of the most critical principles of PAR is creating and sustaining partnerships in which all members share control of the decision-making process, to recognize that researchers and participants have both situated and experiential knowledge that can benefit each other (Minkler and Wallerstein, 2003; Karnieli-Miller *et al.*, 2009). However, in many contexts, ‘compliance cultures’ in leadership result in passive following of rules at sub-national levels rather than active participation in decision-making losing the transformative potential of bottom-up approaches (D’Ambruoso *et al.*, 2019). In addition, there are some beliefs that community members/non-health participants do not know anything about health and so should be told what to do can be present.

**Figure 4:**
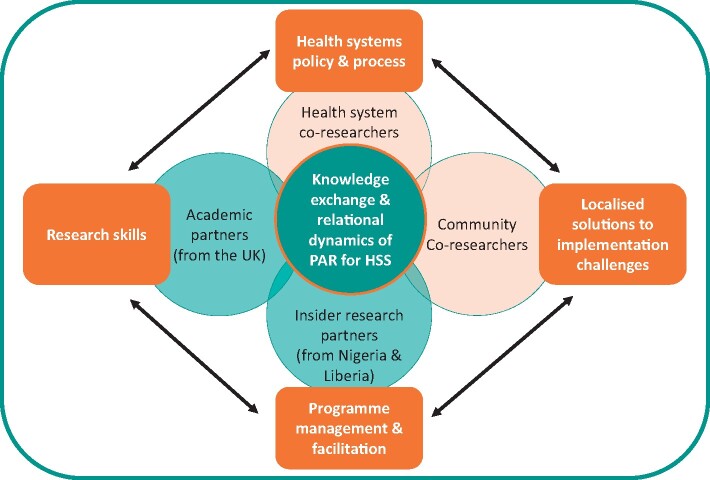
Knowledge exchange and relational dynamics of PAR for HSS

This principle was developed after consideration of the inter-relational dynamics between communities, HSAs, academic researchers and inside research partners. By critically examining equity between the different levels of partnerships, we challenged ourselves to be reflexive of our own and others practice, a well-documented way to add quality to PAR projects. Figure 4 below highlights the inter-relational dynamics of the various partners within the PAR project and the knowledge base that each partner brought to the research. Community co-researchers referred to in Figure 4 are members of the community that took part in participatory research methods to share problems and explore solutions to NTD programme challenges. Below are some descriptions of this process with practical examples of how knowledge exchange between partners had to consider context, culture and historical relations.

#### Examples of negotiating existing health systems power differentials including buy-in to a bottom-up approach

Initially existing health systems structures and historical ways of working meant that there was resistance to bottom-up PAR approaches. PAR aims to challenge such structural barriers and present opportunities for local level actors to add innovation and creativity to localized problems.

The importance of creating a safe, enabling environment for dialogue, helped to ensure that perspectives from all levels in the system were captured through research and implemented through actions, one HS Co-R explained this;

*they took the bottom-up approach…a collaborative research process that enables every participant to talk freely, creates an enabling environment for them to relax, feel free to speak …* (HS Co-R, Nigeria).

The bottom-up approach which ensured inclusivity of community and local level implementers was reported as unique and new;

*I think the way it was disseminated was just unique because it was better to take the information from the lower level to the higher level, then you will definitely impact change* (Research lead, Liberia).

The approach was well received across the health systems and was promoted during reflective meetings and dissemination of the research findings by co-researchers, however it took time to develop acceptance.

*… the voice of everyone is taken into consideration to make a decision of how the program is to be implemented… not just a decision that is being made at the top level* (Research lead, Nigeria).

Solutions and ideas from local level HSAs were used to shape discussions with national and sub-national stakeholders. This supported reflection on practice with many implementers enjoying the innovation they observed at community level. HS Co-Rs highlighted that engagement through participatory methods also developed soft skills such as ways to communicate with communities, how to elicit trust and provide an environment for them to openly share their experiences and come up with solutions.

*…The manners [ways] of approaching people, I think that is one of those things that already I have it, but it was increased* (HS Co-R, Nigeria).

Learning packs and the communication strategy (COUNTDOWN Nigeria, 2018; Zawolo *et al.*, 2018; COUNTDOWN Consortium, 2019;  Ministry of Health-government of Liberia & COUNTDOWN Liberia)  which synthesized community evidence were helpful in ensuring that different community ideas were captured and continuously referred to by multilevel stakeholders.

#### Factors found to influence equitable partnerships between UK researchers and Nigeria/Liberia researchers

Health systems research in LIMCs often involves the participation of partners from an international (often high-income) context (Dean *et al.*, 2015). When using a PAR approach, we are aiming to achieve equitable research partnerships, however identifying ways to communicate can be challenging;

*… every year, we try to review our communication…to know the issues and how to resolve it especially in terms of communication…it has encouraged exchange of ideas, exchange of skills as we try to build each other …* (Research lead, Nigeria).

In Liberia, the difference in the PAR process compared with other research reflected the establishment of more equitable research partnerships;

*[Many] provider organisations … employ Liberians to do the research and after the research is completed, they pack up and leave …. But that is unlike [this] project where the Liberians are considered key partners, they are involved in key decision making …* (Research lead, Liberia).

However, a critical reflection from Liberia was that strengthening capacity should be equitable and take a team approach;

*I think that the capacity building was not equitably distributed…The concentration should not just be on specific individual…I’m just saying that there are certain people whose capacity is built far more than other people* (HS Co-R, Liberia).

The research team reflected that more needs to be done to develop strategies for group capacity strengthening, particularly in contexts of fragility where research capacity is often reduced. This includes finding the time to strengthen capacity of co-researchers who have also busy jobs within the HS.

Equitable and transparent decision making between research partners and an appreciation of each partners’ strengths is an essential component in the development of international collaborations aimed at multi-directional (north–south and south–north) capacity,HSS (Dean *et al.*, 2015) and informed partnership processes.

*I would think that part of that is understanding the context of how we work. It’s a benefit because it adds to your skills and knowledge about the next thing you want to do in the context of low-income countries like ours …* (HS Co-R, Liberia).

Ensuring equity in research outputs is also essential in shaping equitable research partnerships (Dean *et al.*, 2015; Abimbola, 2019). In Nigeria, the research leads expressed that capacity had been built at all levels, including through joint publications, other research collaborations and through long-term research relationships that have been established.

*Everybody in one way or the other has gotten better ideas or better approach on how to do things … it has increased our network of friendship and people to work with. It has helped us to develop our career better, learn new things and skills in every aspect* (Research lead, Nigeria).

### Principle 4: Embed relational practices that lead to new political forms of participation and inquiry within health systems

#### Links to existing PAR principles

This principle concerns relational dynamics and practice, and critical awareness of these through a reflexive process amongst HSAs and within health systems. Globally action researchers highlight that questions around quality and validity should include quality of interaction during the inquiry and the resulting political forms that have been challenged and changed to sustain the intervention and ongoing practice (Reason and Bradbury, 2001). Have the values of democracy been actualized? What is the relationship between initiators and participants over time? What are the long-term implications for infrastructure and political forms within health systems?

When considering the quality of interaction between HSAs we found that there was new political forms of participation that had arisen through taking part in PAR. The PAR process facilitated new relational practices, all of which pertained more to HSS that are not explicitly included in other PAR principles, hence the introduction of this new principle. Below we share evidence from our analysis to support this.

#### Examples of how researchers navigated relational, political and social complexities

The research leads in Liberia and Nigeria had to carefully navigate political and social complexities that are inherent within health systems as social institutions (Sheikh *et al.*, 2014). To do this they had to build strong relationships while continuously assessing their role and positionality, negotiate power within the system and communities and carefully expose bottlenecks that hierarchy poses to implementation challenges.

*… if this state and local government implementers do not find you approachable… the researchers suffer… we ask them questions how they would want things to be done and they share their fears, their concerns with us … I have a very good relationship with the [HSA] … she calls me too with a personal level, we discuss, we relate, and then we are able to work better when it gets to the [research]…* (Research lead, Nigeria).

The research leads were rewarded for their efforts as HSAs gradually transitioned from supporting staunch hierarchy to advocating for the bottom up/participatory approach used in the research. A research facilitator reflected on how the State Co-Rs realized the value in sharing ownership of the planning with local level implementers:

*I compare this [action planning] meeting… to the mid-term stakeholders meeting … with a lot of dominance, from the state … by the second day I saw them begin to … ‘Ok now let’s sit down, we need to plan to ensure that what happens is what they [communities] say they want.’ …. That’s significant for me because now they are letting go of their power and empowering more, the people at the local level* (Reflexivity session with research team, Nigeria).

A local level implementer also reflected on being involved in planning and how this differed from previous NTD treatment cycles:

*There are difference, for 2017 …, before once the state is ready they just tell us to implement it even without bringing us in, but this time around, we sat down and planned how to go about the sensitization and other activities* (Local level HSA, Nigeria).

Developing partnerships and strengthening links between HSAs were identified as a learning point from being part of the PAR process which added quality to the research. The research leads successfully communicated the values of democratic participation, however it was time and resource intensive and challenged researchers’ views of their own practice;

*…I keep trying to evaluate my role as researcher facilitating a PAR process …I’ve had to tell people what to do, sometimes I’ve wondered, at other times, if I was not playing more of an implementer role than that of a researcher* (Research lead, Nigeria).

It is important to recognize that some of the language used within interviews (e.g. ‘I would tell them … we should respect’, or ‘you allow them …’) could be interpreted as autocratic and not aligned with a participatory worldview. Mitteness and Barker, (2004) highlight that researchers must accept that a common ground is only ever fleeting and that social hierarchies exist and cannot be modified by ideological stances. In this case the principles of PAR are reflected in actions and dialogue by HSAs, but cultural communication will still inherently be engrained within conversation and takes time; open, honest and trusting communication and reflexivity to redress. Thus, culturally appropriate best practices supporting participatory facilitation will continually be realized over time. The research leads in Nigeria and Liberia are a key part in this process as facilitators and their task is not an easy one.

The extent of the facilitation role in this process was not grasped initially, it was realized over time. PAR projects that span across communities and health systems would benefit from having this knowledge up front and considering how this may impact the project. Furthermore, research leads within LMICs are often new to PAR and so their own understanding and learning is gradual and shaped by the context in which they are working. Many deliberations and reflexivity sessions between UK academic researchers and research leads in Liberia and Nigeria took place about what their role was; this was a learning process for us all. The research leads shared concerns of influencing the research outcome or being looked upon as programme implementers and managing the ‘messiness’ of the research process;

*… this one [PAR] is ‘I am guiding you on the road, but I really don’t know where it is going to get us to. But I know that we are going somewhere!’ And when we arrive there it’s like ‘oh, ok! So this where we are all going!*’ (Research lead, Nigeria).

Regular audio and written reflexivity through text chat, Skype and in person took place to jointly decide how to manage the facilitation role, ensuring quality of research findings whilst maintaining equitable partnerships with stakeholders and co-researchers. The time and commitment to reflexivity is vital to manage the complexity of PAR approaches for HSS as found in other co-production projects for health policy and systems research (Gilson *et al.*, 2011; Lehmann and Gilson, 2015; Ozano and Khatri, 2018).

### Principle 5: Develop structures for ongoing learning at multiple levels of the health system

#### Links to existing PAR principles

PAR approaches strive for long-term impact through expanding a knowledge base that will contribute to change (International Collaboration for Participatory Health Research, 2013). For systems-level change, health systems, as complex adaptive systems, need to continuously adapt, learn and apply new knowledge to current challenges. Recognition of the importance of learning from experience allows for understanding the intricacies of systems functioning and co-design of actions for implementation challenges (Swanson *et al.*, 2012). This requires establishing trust and demonstrating quality and validity of findings and solutions to implementation challenges (Springett *et al.*, 2011; Lehmann and Gilson, 2015; D’Ambruoso *et al.*, 2019).

This principle was derived after numerous reflections from interviewees related to capacity strengthening throughout the project and sustainability of the PAR approach and new changes that resulted from ongoing and embedded learning from data collection through to dissemination.

#### How learning was shared and embedded within health systems

PAR which has also the aim of HSS through changes to planning, policy and programme implementation requires outputs that can be disseminated to HSAs not directly involved in the research process. The co-production of draft outputs and tools to help scale-up intervention changes, such as participatory guides for planning, learning packs, visual representation of findings and policy briefs all served as useful mechanisms for co-researchers to engage wider stakeholders (Lavis *et al.*, 2009; Adekeye *et al.*, 2017; Zawolo *et al.*, 2018, 2019). Evidence-informed health policy development requires mechanisms that equip policy makers with the tools to support change, this includes accessible breakdown of evidence produced from the research and the impact that it will have (Lavis *et al.*, 2009). A critical strength of these two projects was the involvement of HS Co-Rs who presented findings, openly discussed what they learned during the research and suggested how findings can be scaled up within the wider programmes. Co-researchers are ideally placed to have such policy dialogues as they have been part of evidence collection, observed the outcome and know resources are required. They can disseminate information to policy makers in terms that researchers may not. Having joint ownership of research process and outputs with HSAs supports the integration of scientific findings in policy implementation and HSS (Vanyoro *et al.*, 2019).

#### Examples of how the PAR approach could be sustained along with new soft skills associated with the democratic principles and ethical standards of co-producing knowledge

One of the main themes that emerged from the data was capacity strengthening. In the two contexts it is common within the health sector for HSAs to transfer into new roles. Understanding participatory approaches and ethical standards, which help explore implementation problems and solutions with communities, is a transferable skill.

The formal training in Nigeria was perceived as a good way to prepare for the research, particularly in relation to ethical conduct;

*… It equipped us with the right skills and the knowledge we needed for the work … the dos and don’ts, the ethical requirements … basically to avoid unethical conducts on the field …* (Research lead, Nigeria).

Strengthening capacity to be more responsive to field observations and problems identified during field visits was highlighted by co-researchers as one of the lessons learned from being part of the PAR process;

*My roles, whatever I see on the field that is going to be useful for the program, I disseminate it to the DPH [Director of Public Health] and we incorporate those useful things into the program so the PAR has really helped us a lot in bridging gaps we have in the program* (State Co-R, Nigeria).

This learning process was described by a research lead as a snowballing effect of learning:

*… the implementers … go back and share experiences … even with their superiors at the implementation level, a lot of information exchange has occurred … you can see that it’s like a snowballing … it’s like the information is going from one person to another* (Research lead Nigeria).

Knowledge exchange and capacity strengthening that occurs from engaging in a PAR process adds another level of shared learning that should not be ignored. Once the principles of PAR are learned they have value within the health system;

*… the idea of having state implementers involved in the research has always given some guarantee of sustaining the program and the moment we are able to get the policy makers to buy into this approach of participatory action research, then it's a done deal… they could see the benefit and they see that it is an effective approach …* (Research lead, Nigeria).

Supporting and equipping HS Co-Rs to be able to train colleagues on new skills should be an obligation of research teams. In Nigeria, a research lead explained the importance of sharing learning for sustainability;

*… now that we have gone through this first [PAR] round, is to go through the second round with less researcher involvement and with the implementers … in driving the process more … for them to learn the ropes of planning, carry out the plans themselves, evaluate the plans themselves and see where they can make the difference. So, empowering them will provide that sustainability* (Research lead, Nigeria).

#### Sustainability of new co-produced knowledge to instigate policy and programmatic changes

The interviews highlighted that programmatic improvements observed could partly be attributed to implementers knowing they were being observed by the research team. Therefore, the next phase will be to extract researchers from the process. To do this, guides and tools will be key to equipping Co-Rs with the skills to scale up the process. Co-developing outputs with synthesized research findings and sharing these with those who have power to make permanent change was part of the process in each country context. One Local level implementer stressed the importance of having products that capture learning to share with others and how this is critical in contexts with regular staff changes;

*Even if I leave the local government, it’s [learning pack] not my property, it’s a thing I can hand it over to my successor. The only thing is that they should extend it to other local government* (Local level HSA, Nigeria).

In Liberia, a HS Co-R spoke about the importance of considering where the budget and resources will come from to sustain changes to the programme or to implement policy;

*… so politicians know that money has to come from the top—that’s the only way it stands up* (HS Co-R, Liberia).

One of the challenges raised regarding sustainability of change is the monitoring after the project ends;

*Maybe the challenges will be centred around monitoring the process. Who do we have to monitor the process and make sure it is fully implemented?* (Research lead, Liberia).

At the local level, implementers stressed the importance of ensuring the project outputs reach local levels for sustainability;

*… this has never been done since this program started until this year 2018 … but you know if at the end we will not get any write-ups for health workers to know what has been done during the research, we will not feel it* (Local level HSA, Nigeria).

Although the negotiations of scaling up findings in each country are still ongoing, key steps identified are shown in Box 3. All of which have started to create structured spaces for policy change dialogue and to develop communities of practice as health systems realize the value in the approaches used (D’Ambruoso *et al.*, 2019).

**Box 3**

Ways to support ongoing and sustained learning for policy and planning changes

- Equip HSAs with the tools and skills to share learning with colleagues after the project ends
- Build in and capture reflexivity opportunities throughout the project using a mixture of individual and group techniques
- Establish multilevel working groups with stakeholders to generate ownership of tools and outputs and to make context appropriate decisions about scale up
- Support co-researchers to present the process and outcomes at national/regional steering committees
- Continue to engage with communities and work out ways that they too can be involved in multi-level working groups and scale-up processes, e.g. through the engagement of village health committees.
- Engage with health actors outside of the programme on the PAR process
- Co-design capacity strengthening sessions to develop soft skills involved in using participatory research for understanding community context and working more equitably with lower levels of the health system
- Encourage co-researchers to share learning in different forums
- When presenting outcomes, also capture and share lessons from the process

### Limitations

One of the limitations of this research was the lack of inclusion of community level stakeholder perspectives. This article reflects on PAR from the view of health systems stakeholders with subsequent manuscripts prioritizing community experiences. However, community co-researchers were central to the PAR process and took part in participatory research methods to identify problems and propose solutions which the health system was able to implement within resource restrictions [see supplementary files and online learning packs (COUNTDOWN Nigeria, 2018)].

In addition, within Liberia, the relationships for implementing action plans within the PAR process were still in development and may add to the guiding principles when they complete the first PAR cycle. However, the five principles identified from this research will support quality and reflections within the next stages of the PAR cycle.

## Conclusion

Understanding of the core principles together with clear documentation of the process is vital for ensuring quality and ethical standards within the implementation of PAR. This is only possible through extensive reflexivity which takes time, resources and commitment (Finlay and Gough, 2008; Forman, 2015; Ozano and Khatri, 2018). Through reflection on and application of existing PAR principles and the identification of five additional principles we have facilitated a means of exploring; HS relational dynamics, the processes that support systems and policy change, and some of the barriers and enablers that facilitate ethical democratic HSS overall. This research has drawn on multi-country findings to develop five guiding principles for ethical standards, quality and ongoing learning in implementation research that utilizes a PAR framework and aims to strengthen health systems (Figure 1). However, more research is required to understand and develop conceptual thinking around the intersection between PAR and HSS. International bodies for participatory research have a key role to play in facilitating more communities of practice between researchers and implementers using participatory approaches to strengthen health systems in LMIC and to open up opportunities for cross country learning (Ozano, 2017).

Whilst many PAR projects are contextually bound, due to the application of local knowledge, we have been able to present shared learnings from two diverse contexts that could be useful in guiding other programmes aiming to strengthen health systems and develop sustainable programmatic improvements. Despite having very diverse socio-political histories and health systems organization or infrastructure, we found that understanding best practices in navigating issues of power and participation to establish equitable, quality and sustainable partnerships for PAR were common, supporting the generalizability of these principles to other LMIC settings.

## Funding

This project was funded by COUNTDOWN project (Grant ID—PO 6407) which is a multi-disciplinary research consortium dedicated to investigating cost-effective, scaled-up and sustainable solutions, necessary to control and eliminate the seven most common NTDs by 2020. COUNTDOWN was formed in 2014 and is funded by UKAID, part of the Department for International Development' with UK Foreign, Commonwealth and Development Office (FCDO) Grant number 6407. This article is part of the supplement ‘Innovations in Implementation Research in Low- and Middle-Income Countries’, a collaboration of the Alliance for Health Policy and Systems Research and Health Policy and Planning. The supplement and this article were produced with financial support from the Alliance for Health Policy and Systems Research. The Alliance is able to conduct its work thanks to the commitment and support from a variety of funders. These include our long-term core contributors from national governments and international institutions, as well as designated funding for specific projects within our current priorities. For the full list of Alliance donors, please visit: https://www.who.int/alliance-hpsr/partners/en/.

## Notes

*Ethical approval.* All research processes were ethically approved by; the National Health Research Ethics Committee of Nigeria (NHREC/01/01/2007-19/12/2017); the University of Liberia Pacific Institute for Research and Evaluation Ethical Review Board (UL-PIRE IRB -16-09-009 (CR) and the Liverpool School of Tropical Medicine research ethics committee review board (17-065). Informed consent was obtained from all participants. Some of the research participants are also authors of the paper as they are academic researchers within the PAR programmes.
